# Short-Term Morphological and Quantitative Changes in Non-Exudative Macular Neovascularization Using Spectral-Domain OCT and OCT Angiography: A Pilot Study

**DOI:** 10.3390/jcm14113622

**Published:** 2025-05-22

**Authors:** Mariachiara Di Pippo, Daria Rullo, Elisa Maugliani, Andrew John Lotery, Solmaz Abdolrahimzadeh

**Affiliations:** 1 Ophthalmology Unit, Neurosciences, Mental Health, and Sense Organs (NESMOS) Department, Faculty of Medicine and Psychology, University of Rome Sapienza, 00189 Rome, Italy; mariachiara.dipippo@uniroma1.it (M.D.P.); daria.rullo@uniroma1.it (D.R.); elisa.maugliani@uniroma1.it (E.M.); 2 Clinical and Experimental Sciences, Faculty of Medicine, University of Southampton, Southampton SO16 6YD, UK; a.j.lotery@soton.ac.uk; 3 St. Andrea Hospital, Via di Grottarossa 1035/1039, 00189 Rome, Italy

**Keywords:** non-exudative macular neovascularization, quiescent macular neovascularization, age-related macular degeneration, Image J, image processing, optical coherence tomography angiography, spectral-domain optical coherence tomography, choroidal vascularity index, retinal imaging

## Abstract

**Background/Objectives:** The aim of the current investigation was to assess the short-term changes in retinal-choroidal vasculature and the morphological complexity of non-exudative macular neovascularization (NE-MNV) using optical coherence tomography angiography (OCTA) and spectral-domain optical coherence tomography (SD-OCT). **Methods:** Sixteen eyes of 12 patients with NE-MNV underwent baseline and six-month follow-up examinations, including comprehensive ophthalmological assessment and imaging. Central macular thickness, foveal avascular zone, vessel density, flow area, and choroidal vascularity index were analyzed. NE-MNV morphology was quantitatively assessed for area, vessel characteristics, and fractal dimensions. **Results:** Significant changes in NE-MNV morphology were noted over six months, especially in fractal dimensions, vessel junctions, and vessel length (*p*-values: 0.01, 0.037, and 0.036, respectively). While there was an increase in the NE-MNV area, it did not reach statistical significance. No significant changes were shown regarding the standard SD-OCT and OCTA output parameters or choroidal measurements. **Conclusions:** The increase in NE-MNV fractal dimensions suggests rising complexity in the neovascular network and may indicate possible implications for clinical management. The correlation between baseline and follow-up measures underscores a trend toward complexity, pointing to the necessity for closer monitoring of patients with higher NE-MNV fractal dimensions.

## 1. Introduction

Age-related macular degeneration (AMD) is the principal cause of severe vision loss in the developed world, with a continuously increasing occurrence. In the EU, 70 million individuals are affected, and this number is expected to increase by 18% by 2050 [1].

AMD is characterized by central vision loss due to structural and vascular alterations within the choriocapillaris, retinal pigment epithelium (RPE), and the outer retina. The pathogenesis of AMD involves a spectrum of biological pathways, including complement activation, lipid metabolism, and oxidative stress. These factors collectively pose a challenge in accurately predicting disease progression and customizing patient-specific treatments [2]. AMD involves alterations in the RPE, marked by the onset of modifications in its layer and the accumulation of drusen in early and intermediate AMD stages. Late AMD is either characterized by geographic atrophy (dry AMD, representing 80–90% of cases) or the development of a macular neovascular network (wet AMD, constituting 10–20% of cases) [3,4]. Wet AMD, in particular, is noted for the development of macular neovascularization (MNV), which can be classified according to their origin in type 1 neovascularization, involving neo-vessels sprouting from the choriocapillaris that reach under the RPE through Bruch’s membrane; type 2 neovascularization, located within the subretinal space; and type 3 neovascularization, also known as retinal angiomatous proliferation, where new vessels originate both from the neuroepithelium and from the choriocapillaris [5,6]. The presence of MNV, regardless of its origin, can lead to the formation of intraretinal or subretinal exudation, necessitating targeted therapies to absorb the exudation and reduce neovascular activity. Targeted anti-vascular endothelial growth factor (anti-VEGF) therapies have partially addressed this need, and represent the current treatment of choice [7].

Recently, non-exudative macular neovascularization (NE-MNV) has gained attention in the field of AMD research owing to the development and widespread availability of improved imaging systems. This was initially described by Querques et al. as “quiescent choroidal neovascularization”, characterized by the absence of any indications of exudative activity and, specifically, no intra- or sub-retinal fluid accumulation for at least six months. Lesions at spectral-domain optical coherence tomography (SD-OCT) appear as moderately reflective material under the RPE, causing uneven elevation. They exhibit late-stage hyperfluorescence in fundus fluorescein angiography (FFA) without leaking or pooling, as well as late-stage hypercyanescence in indocyanine green angiography (ICG) that outlines the neovascular formations [8]. In recent years, multiple investigations have conducted a comprehensive evaluation of these lesions, resulting in various proposed terminologies—including “quiescent”, “subclinical”, and the more recently suggested “non-exudative” [9]. Current research has been looking at the effectiveness of optical coherence tomography angiography (OCTA) as a non-invasive imaging tool for examining NE-MNV in alternative to invasive traditional angiographic approaches such as FFA and ICG. OCTA represents a promising resource not only for the diagnosis of NE-MNV but also for the research of potential biomarkers related to these lesions that may suggest an evolution or progression of the disease [10,11]. In this context, Deshpande et al. recently proposed a method of processing OCTA images within an open-access software (Image J, Fiji) in myopic CNV, which provides a skeletonized image of the neovascular network and allows a quantitative analysis of lesions [12].

The aim of the current study was to evaluate OCTA and SD-OCT characteristics of retina-choroidal vascularization and the morphological and structural complexity parameters of neovascularization in NE-MNV over a six-months period. The primary goal was to determine any changes among SD-OCT/OCTA, choroidal, and neovascular parameters at baseline and at six months of follow-up for quantitative and qualitative analysis of retina-choroidal vascularization in the evolution of NE-MNV. The secondary objective was to identify potential biomarkers predictive of an evolutionary activation process of the NE-MNV.

## 2. Methods

This pilot observational longitudinal study was conducted at the Retina Centre of the Ophthalmology Unit, Department of Neurosciences, Mental Health and Sensory Organs (NESMOS), University of Rome Sapienza, Sant’Andrea Hospital. The study aimed to explore short-term morphological and quantitative changes in NE-MNV in a small cohort as preliminary data for future larger-scale investigations.

A total of 16 eyes from 12 subjects with treatment-naïve NE-MNV were included. The study protocol was approved by the Institutional Review Board of the University of Rome Sapienza and adhered to the tenets of the Declaration of Helsinki. All participants provided informed written consent.

In order to be included in the study, individuals required SD-OCT and OCTA evidence of treatment-naïve NE-MNV without any signs of intra- or subretinal exudation for at least six months and clear optical media in order to acquire adequate SD-OCT and OCTA scans. Exclusion criteria were exudative MNV, previous intravitreal anti-VEGF injections in the last year, more than 4 Diopters spherical equivalent, intraocular pressure exceeding 18 mmHg or glaucoma, diabetic or hypertensive retinopathy, presence of epiretinal membranes using SD-OCT.

Subjects underwent comprehensive ophthalmic evaluation, including visual acuity measurement using the Early Treatment Diabetic Retinopathy Study (ETDRS) charts, spherical equivalent calculation for refractive error, anterior segment examination through slit-lamp biomicroscopy, intraocular pressure assessment using Goldmann applanation tonometer, and fundus assessment after pharmacologically induced mydriasis. SD-OCT and OCTA scans were carried out at baseline and at six-months. Choroidal vasculature and non-exudative macular neovascularization were quantitatively analyzed using the open-source software Image J (Fiji, version 2.9.0). The imaging was carried out and reviewed by two expert investigators masked to the timepoints.

### 2.1. OCTA Parameters and CVI Calculation

OCTA imaging was performed using the Solix AngioVue Retina^®^ instrument (Optovue Inc., Freemont, CA, USA) with scan dimensions of 6.4 mm by 6.4 mm. The incorporated AngioAnalytics™ software of the instrument quantified the foveal avascular zone (FAZ), vessel density (VD) of the superficial and deep capillary plexus (SCP and DCP), and choriocapillaris flow areas. The VD represents the percentage of pixels that indicate vessels in the total pixel population for the SCP and DCP of the retina. Measurements of the outer retina and choriocapillaris flow area were performed on an automated 2 mm diameter circular area centered on the fovea. Scans were assessed for proper segmentation and clear flow signal. If motion artifacts were detected, indicated on the enface scans by dark lines, the scan was repeated. OCTA scans were also compared with SD-OCT cross-sectional scans and enface images to identify potential shadowing effects on the choriocapillaris.

The SD-OCT raster scan mode using a subfoveal B-scan image was used to calculate the choroidal vascularity index (CVI). Utilizing Image J software (Fiji version 2.9.0.), the luminal choroidal area (LCA) and the total choroidal area (TCA) were measured, and these values were used to calculate the CVI, based on the technique of Sonoda et al. [13,14,15]. In detail, B-scan OCT raster image was exported into Fiji software, the “straight line” tool was used to set a 12 mm scale. Then, using the “rectangle” tool, a region of interest (ROI), a 1500 µm area centered on the fovea, was marked. Within the ROI, the “polygon” tool outlined the choroidal area, bounded superiorly by the choroid-RPE junction and inferiorly by the choroid-scleral junction, to calculate the TCA. Subsequently, the “oval selection” tool was used to adjust the image brightness based on the average value of three large choroidal vessels. Following the conversion of the image to 8-bit, Niblack’s autolocal thresholding was used for image binarization, initially visualizing it in only black and white pixels. Then, through conversion to a color image, the LCA and the stromal choroidal area (SCA) were delineated. The ratio of the LCA/TCA determined the CVI.

### 2.2. Quantitative Non-Exudative Macular Neovascularization Assessment

**Figure 1:**
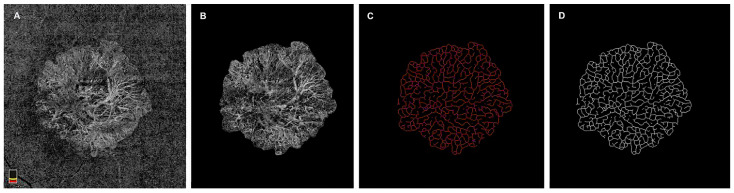
Non-exudative macular neovascularization (NE-MNV) image processing: (**A**) Optical coherence tomography angiography enface image of NE-MNV; (**B**) manually delineated NE-MNV on Image J software; (**C**) AnalyzeSkeleton software output of NE-MNV; (**D**) Binary skeletonization of NE-MNV image.

The scans were processed using Image J software (Fiji version 2.9.0.). Deshpande et al. developed a macro that computes nine OCTA metrics—vessel area, macular choroidal neovascular (mCNV) area, vessel density, vessel length, vessel junction, fractal dimension, tortuosity, vessel diameter, and junction density. In parallel, the accuracy of these automated metrics was validated against a manual analysis by Wang et al., showing results consistent with expected ranges [12,16]. In the method, prior to skeletonization, a pre-processing phase is necessary. The macro uses expert-labeled retinal OCTA images, which must undergo manual delineation to ensure that measurements focus exclusively on the mCNV lesion. The resulting cropped images are then converted to 8-bit grayscale to facilitate the skeletonization step. Contrast and brightness adjustments are also recommended to reduce image noise. The processing pipeline follows a linear sequence. First, a Gaussian Blur filter with a decay radius of 1 is applied to reduce noise. Next, a Hessian-based Frangi filter exploits the Hessian eigenvectors to evaluate the likelihood of vessel presence in the image. An 8-bit binary image is then produced via Auto Local Median Thresholding, in which thresholds are computed for each pixel based on the local grayscale median across an 8-pixel radius. From this binary image, the statistical area is used to derive the vessel area, which, combined with the mCNV area, yields the vessel density. Continuing the workflow, a Mexican Hat Filter with a 13-pixel radius (determined empirically) is applied, producing a skeletonized binary OCTA image. This skeletonization allows the extraction of vessel length, junctions, fractal dimension, and tortuosity. In turn, vessel diameter and junction density are derived from these computed parameters. Finally, the skeletonized 8-bit OCTA images furnish detailed information on mCNV lesion vascular activity. The “AnalyzeSkeleton” Image J plugin generates a statistical assessment of the branching morphology by removing isolated vertices and branches that terminate at single endpoints. This process facilitates determination of vessel length, tortuosity, junction count, junction density, and vessel diameter. Vascular complexity is quantified through the fractal dimension, calculated by the box-counting method. Once these steps are complete, all nine OCTA biomarkers have been evaluated. The images obtained through the aforementioned process are then imported into the “Input” directory folder, and the “Latest safe” plugin is executed, yielding the parameters of interest (Deshpande et al. 2023, Wang et al., 2021) Figure 1 shows NE-MNV image processing [12,16] (Figure 1).

The following list describes the various quantitative features of neovascularization in detail:

- MNV Area (mm^2^): This reflects the size of the neovascular area.
- Vessel area (mm^2^): This is a biomarker of the vessel dimensions, specifically the vessel components where active flow signals are detected within neovascularization.
- Vessel density: This refers to the vascular density and is the ratio of vessel area over MNV area.
- Vessel Length (mm): This represents the total length of the neovascularization.
- Vessel junctions: This denotes the number of vascular junctions and serves as an index of the neovascular structure internal branching, identified as the connection points within the MNV vasculature.
- Junction density (n/mm): This is the ratio between number of junctions over total neovascular length, reflecting the complexity of the branching.
- Vessel tortuosity: This is a morphological marker that quantifies the micro-tortuosity of the MNV.
- Fractal dimensions: This is a biomarker that characterizes the complexity of the MNV, evaluating the intricacy of even very small vascular networks.

### 2.3. Statistical Analysis

As this was a pilot study, no formal sample size calculation was performed. The small sample was chosen to explore feasibility and preliminary trends in morphological and quantitative changes associated with NE-MNV. Consequently, the study is limited in statistical power, and the results should be interpreted with caution.

Data were expressed as mean ± standard deviation or median and interquartile range for continuous variables, and the number of cases (and percentages) for categorical variables. All variables were tested for normality using the non-parametric Kolmogorov-Smirnov test. The differences in the distributions of the variables were assessed using a paired Student’s *t*-test. Categorical variables were evaluated using the χ-square test or Fisher exact test when appropriate. Pearson correlation was performed to compare variables.

A *p*-value ≤ 0.05 was considered statistically significant. Statistical analysis was performed using SPSS Software (version 27.0, SPSS INC, Chicago, IL, USA).

## 3. Results

The mean age of the patient population was 80.58 ± 7.88 years. There were no statistically significant differences in visual acuity at baseline with respect to the follow-up at 6 months (45.18 ± 9.26 and 45 ± 8.80, respectively).

As regards the quantitative analysis of neovascularization morphology and structural complexity, the differences between means demonstrated a statistically significant increase in the parameters vessel junctions, vessel length, and fractal dimensions at the follow-up examination with respect to baseline. MNV area showed an increase in size at the follow-up, which was however not statistically significant (Table 1).

Table 2 shows mean differences, standard deviations, and 95% confidence intervals (CIs) for the main quantitative outcomes. To further support interpretation, the magnitude of change was assessed: effect sizes were moderate for vessel junctions (0.56) and vessel length (0.54), and small for fractal dimension (0.33), suggesting a potentially meaningful structural change in the vascular network despite the limited sample size.

No differences were found in the analysis of the automated OCTA output values and choroidal vasculature parameters (Table 3).

Correlation analysis showed that increased fractal dimensions at baseline was associated with increased fractal dimensions at the follow-up (r = 0.884, *p*-value < 0.001).

## 4. Discussion

The current study identified a statistically significant change in the quantitative morphological parameters of NE-MNV in the context of AMD over a follow-up period of six months. Specifically, increases in vessel junctions, vessel length, and fractal dimensions of NE-MNV were found. Recent investigations assessed the reliability of OCTA as a non-invasive technique for the study of NE-MNV, in alternative to angiographic methods such as FFA and ICG. Carnevali et al. reported a sensitivity of 81.8% and a specificity of 100% for OCTA in the diagnosis of NE-MNV. Additionally, Faridi et al., in a study involving a sample of 70 patients, found OCTA to exhibit a sensitivity of 100% and a specificity of 97.5% for the same diagnostic purpose. These results contribute to the growing body of evidence supporting the diagnostic capability of OCTA in NE-MNV evaluation [10,17].

A pivotal aspect in the assessment of NE-MNV involves the analysis of biomarkers to determine exudation and, therefore, transition into an active state. In the present investigation, no lesions progressed to an exudative state; however, there was the relatively short duration of the follow-up spanned only 6 months. Indeed, Capuano et al., over a longer period, approximately 45.7 ± 14.7 months, in a cohort comprising 19 patients, reported an exudation rate of 26% [18]. Additionally, Fukushima et al. reported a conversion rate of 30% over two years of follow-up [19], while Carnevali et al. and Serra et al. documented a lower conversion rate of 6% and 8%, respectively, after one year of follow-up [20,21]. Thus, the determination of specific biomarkers for possible exudation in NE-MNV is crucial for the management of AMD. The timely application of anti-VEGF therapy, possibly guided by molecular and imaging biomarkers, could improve visual outcome and enlarge the therapy interval [10,20,22,23]. The present study did not find significant differences between baseline and 6-month follow-up of OCTA standard output parameters or the CVI. It is important to reiterate that none of the patients analyzed in this study experienced exudation, and our findings are consistent with the results of prior research, particularly concerning the central macular thickness (CMT) [8,20,24].

Concerning NE-MNV morphological parameters, various studies reported an enlargement of the neovascular area, notwithstanding the absence of exudative manifestations [10,21,25]. Also in the current study, an expansion of the neovascular area was observed, although the increase did not reach statistical significance. Serra et al. postulated that such augmentation may be attributable to intrinsic pathogenic processes. These authors hypothesized that inactive MNV could serve as a retinal adaptive mechanism, safeguarding photoreceptor integrity against ischemic damage, thereby inhibiting atrophic progression and preserving visual acuity [21]. Consistent with these findings, in our study, the visual acuity demonstrated stability throughout the six-month observational period.

To the best of our knowledge, the quantitative features of NE-MNV using ImageJ software for post-acquisition analysis have not been previously assessed. In our study, among the various parameters examined, the most significant change was observed for the fractal dimensions, a measure that evaluates the complexity and space-filling capacity of vascular ramification [12]. This is particularly noteworthy as it suggests an increased structural complexity within the neovascular membrane network. Increased fractal dimensions could indicate a propensity towards activation, a factor that might have clinical management implications for patients [26,27]. However, the six-month follow-up in this study may be too brief to highlight exudation, and a longer observational study could provide more detailed insight. Additionally, a positive correlation was observed between the baseline fractal dimensions and that at 6 months of follow-up. This outcome might suggest that membranes with greater complexity at baseline are predisposed to develop even more complexity over time. Therefore, it would stand to reason to speculate that NE-MNV with higher fractal dimensions could require more frequent monitoring.

The current study revealed an increase in vessel junctions, a metric that assesses the number of vascular connection points by evaluating the degree of internal branching, as well as an increase in vessel length, which measures the total length of the NE-MNV. The ratio of these two parameters yields the junction density, a value that was found to be elevated in our study, although it did not achieve statistical significance. Takeuchi et al., in a quantitative analysis of patients undergoing anti-VEGF therapy for active CNV, showed a reduction of junction density immediately after intravitreal injection, implying the reduction in branching and the maturation of the vessels that presumably blocked the VEGF pathway [28]. Choi et al. observed an increase in both junction density and vessel length, albeit not significant, in patients with type 1 “unstable” CNV, which required more than three anti-VEGF intravitreal injections over the course of one year. Although the method for calculating the parameters in these studies was not identical to ours, it could be hypothesized that the observed increase in vessel junction and vessel area may indicate an increase in complexity and intra-neovascular network anastomoses, potentially representing a “preparation” for membrane exudation. However, these observations can only be tentatively confirmed through longitudinal observation of the NE-MNV with a more extended follow-up period.

The present study was subject to several limitations. Notably, this was a preliminary investigation with a limited sample size and a short follow-up period. Traditional invasive imaging using FFA and ICG was not performed, although recent studies have demonstrated high sensitivity and specificity of OCTA. Furthermore, SD-OCT and OCTA scans were independently verified by two experienced investigators, including the review and manual adjustment of image segmentation where necessary. While this ensured consistency, the manual nature of the segmentation and subsequent image processing using Image J may still introduce potential sources of bias, particularly due to the lack of formal intergrader agreement assessment, which should be taken into account when interpreting the results.

Further studies are recommended, employing a larger sample size and examination of the long-term longitudinal changes in morphological NE-MNV parameters, assessing their behavior in potentially indicating transition from quiescent to exudative neovascularization. The observed increase in parameters such as vessel junctions, vessel density, and fractal dimensions at follow-up may reflect underlying biological processes that are significant for disease progression and treatment response. Moreover, it would be of interest to integrate the imaging-based parameters with molecular biomarkers that may provide additional insight into disease activity. In a recent study, Moshtaghion et al. measured VEGF levels in the tears of patients with exudative AMD, finding elevated VEGF levels in the tears of affected patients, which correlated with disease severity [23]. The authors further investigated the origin of VEGF production, whether from the RPE–choroid complex or the lacrimal gland, in an experimental mouse model of AMD, and, notably, they observed that VEGF was primarily produced by the choroid and RPE. Based on these findings, the authors proposed tear VEGF as a potential biomarker to monitor disease activity [23]. In the future, the combined use of molecular biomarkers such as tear VEGF and imaging biomarkers, including the morphological features of neovascular membranes, could become part of routine clinical practice for disease monitoring and prognosis. Additional studies are warranted to probe the clinical significance of such observations and to assess how this information can be leveraged to optimize the management of patients with NE-MNV.

## Figures and Tables

**Table 1:** Morphological non-exudative macular neovascularization analysis at baseline and at 6 months.

Parameters Evaluated	Baseline (n = 16)	Follow-Up (n = 16)	*p*-Value ^a^
MNV area	6.04 ± 5.96	8.42 ± 10.38	0.318
Vessel area	2.67 ± 2.38	2.70 ± 2.39	0.697
Vessel Junction	91.32 ± 126.92	174.37 ± 160.20	0.037 *
Vessel Length	24.99 ± 35.82	46.11 ± 41.81	0.036 *
Fractal Dimension	1.40 ± 0.90	1.43 ± 0.59	0.013 *
Tortuosity	1.15 ± 0.33	1.16 ± 0.54	0.601
Vessel Density	0.480 ± 0.47	0.447 ± 0.11	0.278
Junction Density	3.49 ± 0.59	3.72 ± 0.44	0.153

MNV: macular neovascularization. ^a^ paired *t*-test. * statistically significant (*p*-value < 0.05).

**Table 2:** Statistically significant parameters between baseline and 6-month follow-up.

Significant Parameters	*p*-Value ^a^	Mean Difference	Standard Deviation	95% Confidence Interval	Cohen’s d
Vessel junction	0.037 *	−83.06	145.32	−160.50 to −5.62	0.567
Vessel length	0.036 *	−21.12	36.57	−40.60 to −1.63	0.538
Fractal dimension	0.013 *	−0.03	0.04	−0.05 to −0.007	0.337

^a^ paired *t*-test. * statistically significant (*p*-value < 0.05).

**Table 3:** Optical coherence tomography angiography and choroidal vasculature analysis at baseline and at 6 months.

Parameters Evaluated	Baseline (n = 16)	Follow-Up (n = 16)	*p*-Value ^a^
Foveal avascular zone	0.280 ± 0.12	0.292 ± 0.11	0.702
Vessel density superficial capillary plexus	26.24 ± 4.81	27.78 ± 5.60	0.408
Vessel density deep capillary plexus	31.19 ± 7.05	32.82 ± 7.72	0.483
Flow area outer retina	1.72 ± 0.48	1.76 ± 0.49	0.618
Flow area choriocapillaris	1.53 ± 0.51	1.53 ± 0.55	0.923
Luminal choroidal area	0.968 ± 0.40	1.070 ± 0.49	0.091
Total choroidal area	1.469 ± 0.57	1.604 ± 0.62	0.138
Choroidal vascularity index	0.652 ± 0.38	0.651 ± 0.56	0.896

^a^ paired *t*-test.

## Data Availability

Dataset available on request from the authors.
